# Iodine Status among Somali Immigrants in Norway

**DOI:** 10.3390/nu10030305

**Published:** 2018-03-05

**Authors:** Ahmed A. Madar, Helle M. Meltzer, Espen Heen, Haakon E. Meyer

**Affiliations:** 1 University of Oslo, Department of Community Medicine and Global Health, Institute of Health and Society, Post Box 1130 Blindern, 0318 Oslo, Norway; espenheen@gmail.com (E.H.); h.e.meyer@medisin.uio.no (H.E.M.); 2 Norwegian Institute of Public Health, 0318 Oslo, Norway; HelleMargrete.Meltzer@fhi.no

**Keywords:** dietary determinants, iodine, urinary iodine excretion, Somali immigrants, 24-h urine collection

## Abstract

We lack knowledge about iodine status in the Norwegian population in general, and particularly among immigrants. We aimed to estimate the iodine status and potentially associated factors in a Somali population in Norway. Somali men and women aged 20–73, who were living in one district in Oslo, were recruited between December 2015 and October 2016. Twenty-four-hour urine was collected from 169 participants (91 females and 78 males). Iodine was analysed using the Sandell–Kolthoff reaction on microplates and colorimetric measurement. Information about diet was collected using a short food frequency questionnaire. Iodine intake was calculated from the 24-h iodine excretion. The mean urine volume over 24-h was 1.93 liters (min–max: 0.55–4.0) and the urinary iodine concentration (UIC) varied from 13 to 263 µg/L with a median value of 62.5 µg/L indicating a population with mild iodine deficiency. The median daily iodine intake for the study population was estimated to be 124 μg/day. Mean serum thyroid-stimulating hormone, thyroxine (T4) and triiodothyronine (T3) was 2.1 (SD 1.1) mU/L, 15.0 (SD 2.1) pmol/L, and 5.1 (SD 0.6) pmol/L, respectively. No food groups were associated with iodine intake and neither was gender, age, education level nor length of residence in Norway. In conclusion, this study showed that iodine intake was low, and a considerable proportion of the Somali population studied had sub-optimal iodine status. Monitoring of iodine status should be prioritised and measures to ensure adequate iodine intake, particularly among vulnerable groups initiated.

## 1. Introduction

Iodine is a trace element that is essential for humans. It is a key component of the thyroid hormones thyroxine (T4) and triiodothyronine (T3), which are essential for maintenance of metabolic rate and energy metabolism in adults and play a central role in the growth and development of the brain and nervous system in fetuses and children [1]. Low iodine intake may lead to numerous negative health outcomes, collectively named iodine deficiency disorders (IDD) [2]. IDD are among the most common preventable deficiency diseases globally; they are considered to affect over 1.9 billion people worldwide [3]. The soil in the Nordic regions has by nature little iodine and IDD, including visible goiter, was previously endemic in inland areas where the consumption of saltwater fish was low [4,5]. Fortification of animal fodder began in the early 1950s (2 mg/ kg), and from this followed a heightened concentration of iodine in milk and dairy products, which contributed to the eradication of endemic goiter and the prevention of IDD [6]. Norway is a nation with traditional high levels of milk consumption, and milk, dairy products, fish and fish products have since then provided the main sources of iodine in the diet of Norwegians, contributing with about 80% of total iodine intake [7].

Recommended iodine intake in the Nordic countries and from the World Health Organization (WHO) is 150 μg/day from ten years of age [8,9], while the estimated average requirement (EAR) is 95 μg/day [10]. The proportion of values in a distribution curve of habitual iodine intake that falls below the EAR value will indicate the percentage of the study population that is iodine deficient. More than 90% of dietary iodine intake is believed to be excreted via urine within 24-h, and urinary iodine concentration (UIC) is currently the most practical method for assessing the iodine status in a population [8,11]. The WHO recommends a median UIC of at least 100 µg/L to prevent IDD in the general population [12]. Although casual spot urine samples is the simplest and most common collection method for obtaining median UIC values in a population, 24-h urine collection is often regarded as the “gold standard”. Combined with 24-h urine volume, it provides the basis for estimation of urinary iodine excretion and daily iodine intake. Since 24-h UIC is a pooling of a number of spot urine samples from each individual, covering a full 24 h, we should expect the median of 24-h UIC to represent the same “true” value as the median of casual spot urine samples, only with slightly less uncertainty in the estimates [13].

Generally, there is not sufficient knowledge about the present iodine status in the general Norwegian population and, until recently, IDD were not considered a public health problem. However, mild iodine deficiency seems to have re-appeared in some sub-groups, especially among women of childbearing age, including pregnant and lactating women [14,15,16]. Though we do not know the iodine status of immigrant populations in Norway, a considerable proportion of immigrants come from areas where iodine deficiency is still endemic, and they may thus be vulnerable to developing iodine deficiency if they do not secure sufficient iodine intake through their diet. In other European countries, it has been found that pregnant women and children of immigrant backgrounds are at risk of iodine deficiency [17,18,19]. 

Following the identification of insufficient iodine intake levels in Norwegian pregnant women and the general reduction in milk and fish consumption in the Norwegian population [4], the Norwegian Nutrition Council has recommended evaluation of the iodine status of vulnerable groups [20]. Somalis constitute the largest non-Western immigrant group in Norway (*n* = 41,500) and around 36% (*n* = 15,000) live in Oslo. Generally, the knowledge about the health status in Somalis is limited and data about their iodine status is lacking. The aim of the present study was to evaluate the iodine status and potentially associated factors in a Somali sub-population in Norway.

## 2. Design, Methods and Material

This paper’s data originates from a study assessing risk factors for lifestyle diseases among Somali immigrants in Oslo, Norway, conducted between December 2015 and October 2016. It has a cross-sectional descriptive design. 

The eligible participants were adults of Somali origin, men and women, aged 20–73 years, who lived in the Sagene district in Oslo. This district has one of the highest populations of Somali origin in the city. Collaboration was established with local Somali organizations, a healthy life center and the district medical officer, with all contributing to the planning of the study. Information about the study was shared through the local Somali radio, and community centers in the district. An attempt to contact every adult person with Somali background living in the district was made and those available were invited to participate in the study. Ten youth volunteers and three project assistants identified by partner organizations trained and helped with recruitment and data collection. Pregnant women and anyone suffering from kidney or liver failure, thyroid disorders, cancer and other serious diseases were excluded (*n* = 20). Other exclusion criteria were newly-initiated treatment with diuretics (less than two weeks) and other conditions that make 24-h urine collection difficult. 

## 3. Data Collection

Information about gender, age, education, smoking habits and length of stay in Norway was collected using a questionnaire. Information about diet was collected using a short food frequency questionnaire (FFQ) [21]. The FFQ had previously been used in the Oslo immigrant health study. This was not validated for our study group but was revised and pilot tested in a smaller group of Somalis. 

The FFQ had a semi-quantitative design to capture both dietary habits and intake of dietary supplements. For milk and milk products, frequencies ranging from never to several cups/portions per month, week or daily were noted. For fish consumption, frequencies of <1 time/week, 1–2 times/week, 3–4 times/week, and >5 times/weeks was noted. For egg consumption, the number of eggs consumed ranging from never, 1–3 per week, 4–6 per week, 1 per day or more per day was noted. Frequency of table salt use was collected, although in Norway, few brands of table salt are iodized (maximum 5 μg/g salt), and the food industry is prohibited using of fortified salt [22]. The use of supplements containing iodine, frequency and number of tablets each time taken was noted. 

## 4. 24-Hour Urine Collection

Participants were provided with a urine collection kit with a transparent inspection strip and graduation (urine container of 3.0 L). To assist with urine collection, an additional 500 mL plastic handled jug was provided. Oral and written instructions about urine collection and handling were given participants. An information sheet where the first and last urination time points and any missed voids could be recorded was provided to each participant. The participants were instructed to void their bladders in the first morning of collection and discard this urine, and then collect all urine until and including the morning void of the day after. The containers were kept cool during urine collection, and time for starting, ending and irregularities were noted. The participants were contacted daily by the project assistants to remind them of the urine collection. After returning the 24-h urine specimens, the containers were well stirred, and volume was read and recorded, and two milliliters of urine sample was extracted and stored at −20 °C until analysis of iodine concentration. Only one male participant reported having missed collecting urine once, and he was not excluded from the study on this basis (the 24-h volume was more than 500 mL). 

## 5. Urine and Blood Analysis

All samples were analysed in one batch at the Hormone Laboratory, Oslo University Hospital. UIC was measured using the Sandell–Kolthoff’s reaction (colorimetric) based on the catalytic effect of iodine on the red /ox reaction between arsenic and cerium after ammonium persulfate digestion of the samples. Intra- and inter-assay coefficients of variation (CVs) were ≤8%. The Hormone Laboratory is accredited as a testing laboratory by Norwegian Accreditation according to the standard NS-EN ISO/IEC 17025, with Registration number TEST 099. 

Daily urinary iodine excretion (UIE) was calculated using the following formula:

24-h UIE (μg/day) = UIC (μg/L) × 24-h urine volume (L).

Subsequently, daily iodine intake (μg/day) was estimated using the following formula: Daily 24-h UIE (μg/day)/0.92 [10,23].

The median UIC of the participants was compared with the iodine status criteria developed by the WHO, UNICEF and ICCIDD for adults [12]. 

Participants were given a requisition to take a fasting blood sample at Fürst Medical Laboratory in Oslo [24] and analyses were conducted the same day as blood was taken. Serum Thyroid stimulating hormone (TSH), serum T4 and T3 were determined by the chemiluminiscence method (ADVIA Centaur XP Siemens) with an inter assay coefficient of variation (CV) respectively, 4.6%, 6.5% and 3.2%.

## 6. Ethics 

The study was approved by the Regional Committee for Medical and Health Research Ethics (study code: 2015/1552 REK South-East). Informed written consent was obtained from all participants.

## 7. Statistical Analysis

Analysis of the data was performed using the IBM SPSS statistical software (V.22 SPSS Inc., Chicago, IL, USA). Descriptive statistics are presented as mean with standard deviation (SD) and as medians, with 25th and 75th percentiles for variables that were not normally distributed. To compare variables, relevant statistics such as Spearman correlation analysis and regression models were performed. *p* < 0.05 was considered to be statistically significant.

## 8. Results

A total of 222 participants (112 females and 110 males) aged 20–73 years were included in the study, of whom 169 (76%) returned a complete set of 24-h urine samples. The background characteristics of the 169 participants are shown in Table 1. The background characteristics of the 53 participants who did not collect urine samples were not different from the participants in the analysis (data not shown).

The creatinine values were within the reference range for both genders, where the mean concentrations (mmol/24 h) were 15.5 (SD 4.1) and 10.0 (4.9) for males and females respectively. 

## 9. Iodine Status and Thyroid Hormones

Mean urine volume over 24 h was 1.93 L (min–max: 0.55–4.00) (Table 2). The total 24-h median UIC (25th, 75th percentiles) of all participants was 62.5 (38, 100) µg/L and the median UIC for females was not significantly different from that of males.

Using the WHO reference ranges for defining iodine status based on UIC, the study population should be classified as mildly iodine deficient (Table 3). The median 24-h UIE for the whole group was 114 µg/24-h. Males had slightly higher UIE values than females, but this was not significant. The estimated iodine intake was 124 μg/day for the whole group (Table 2).

Mean concentrations of fT4 was 15.0 (SD 2.1) pmol/L, and mean concentration of fT3 was 5.1 (SD 0.6) pmol/L, all within the reference range. Mean concentration of TSH was 2.1 (SD 1.1) mU/L, while three persons had levels <0.20 mU/L (lowest reference range) and seven persons (4 females and 3 males) had levels above 4.0 mU/L. Generally, there was no association between UIC and any markers of thyroid function (TSH, fT3, fT4). However, two females of the seven persons with elevated TSH had UIC below 20 µg/L. 

The UIC did not differ significantly with respect to the variables gender, age, length of stay in Norway and education level (data not shown).

## 10. Estimated Daily Iodine Intake

We were not able to calculate the iodine intake from diet due to the lack of portion sizes, however, based on the daily UIE, the median daily iodine intake for the whole study population was estimated (on the basis that 92% is excreted) to be 124 μg/day (133 µg/day for males and 114 µg/day for females). The distribution of intake was assessed to determine the proportion of the population below EAR. Around 30% of males and 41% of females had intakes below the EAR of 95 μg/day. The iodine intake was not significantly different between the sexes, age groups, and education level or length residence in Norway. The median iodine intake for the participant that missed one urine collection was 82 µg/day, and when we excluded this from the main analysis, the median daily intake was not different. 

## 11. Food Patterns

Table 4 shows reported consumption of iodine containing foods. Among females, around 2/5 reported not consuming milk and milk products daily. In addition, 39 % of males and 31% of females reported eating fish around three or more times per week. The daily consumption of eggs in both males and females was low. The estimated iodine intake was not correlated with food pattern or any specific food groups. 

### Table Salt and Supplements

Few participants reported using table salt regularly where 6% used it daily and only five persons reported taking iodine-containing supplements daily. Their iodine intake values were not different from other participants. 

## 12. Discussion

This study is the first to evaluate iodine status among Somali immigrants in Norway. Based on 24-h urine collection, the iodine intake findings indicate that the study group of Somalis had insufficient (or sub-optimal) iodine nutrition. Generally, there is little knowledge about the iodine status of the Norwegian population, and particularly about the immigrant population, but our findings suggest that iodine deficiency can be a public health concern for some groups [13,14]. The very few studies on iodine status in European populations that included immigrants focused mainly on pregnant women, and these produced mixed results. Lindorfer et al. reported that a group of women of non-Austrian origin (consisting of 137 women from 37 nations) had higher median UIC compared to women of Austrian origin (105 and 77 μg/L) [18]. The opposite result was seen in Italy, where Italians had a significantly greater median UIC (100 μg/L) than non-Italians (45 μg/L in African and 46 μg/L in Eastern European women) [17]. These differences were explained by different eating habits and consumption of iodized salt [18].

Furthermore, the median UIC in our study is much lower than what was found in a recently conducted study among 617 Somali women of reproductive age (15–49 years) in Somaliland, who had a median UIC of 329 μg/L. The high UIC was explained by the drinking water, which has a high content of iodine in the studied regions [25].

Generally, the average urine volume in adults is estimated to be approximately 1.5 liters/day, which implies that median UIE values (µg/24 h) should be about 50% larger to represent the same iodine status as UIC values (µg/L) [26]. The mean 24-h urine volume in our study is in line with previously published population-based data [26,27,28], however, considerably higher than the 1.5 L. Since the real UIE values capture more information about iodine status than UIC, the volume uncertainty being eliminated, it provides additional information in the judgment of this group’s iodine status. Working backwards, we can say that UIC values should be about 65% of the UIE values. 

The median UIE of 114 µg/24 h in our study corresponds to a UIC of approximately 68–80 µg/L, slightly better than the crude median UIC value of 62.5 µg/L. This indicates that the risk of iodine deficiency is present in this group regardless of the measured or calculated UIC values. 

Furthermore, the large spread in the individual urine volumes in this study is supported by data from other studies [29,30]. Although the participants were informed not to change their daily routines and habits during the urine collection, we are not sure whether our findings represent the habitual urinary volume excretion, or whether participants changed their habits and routines, for example staying home the day they were collecting urine and therefore drank more fluids. 

In Norway, milk, dairy products and fish are the main sources of iodine in the diet. Iodine from milk and dairy products amount to approximately 60–80% of iodine intake. However, in recent years, there has been a significant decline in milk consumption in Norway; many people do not drink milk daily, and around 23% of Norwegian women and 14% of men avoid the consumption of milk [19]. The negative trend in milk intake has direct consequences for iodine intake. In addition, iodine-containing supplements contribute to the iodine intake for daily users. Iodized salt contains a very low amount of iodine and does not contribute much to the iodine intake of those who use this salt. Drinking water, which is a good source for iodine for certain populations globally, contains a very small amount of iodine in Norway (1.7 μg/L) [22]. 

The iodine intake findings of the Somalis suggest lower iodine consumption than the general Norwegian population where the estimated average of iodine intake was 176 and 136 µg/day for men and women respectively [22]. In our study, around 20% of men and 40% of females reported consuming less than 1 cup of milk or dairy products per day, and the consumption of fish, eggs, supplements and iodized salt were low. This seems to be lower than the estimated proportion of milk and milk products consumption of Norwegians (77% of women and 86% of men) [19]. 

In Norway, there is no national iodine supplement recommendation for the general population, and the proportion taking iodine-containing supplements is unknown. In our study, only five persons reported using iodine-containing supplements. Both iodized and non-iodized salt for human consumption are available in the market and most probably only those who are familiar with the differences purchase the iodized salt. 

The intake of milk, dairy products, eggs, iodized salt and the use of supplements have previously been reported to be positively associated with iodine status [23,31,32]. In this study, there was no significant association between dietary factors and iodine excretion. In spite of this, we believe that our findings of low estimated iodine intake could be attributed to relatively low intakes of milk and milk products, fish, seafood, iodized salt, or other fortified foods in our study population. The lack of association between dietary factors and iodine excretion is in line with what found in another Norwegian study [33], but contrary to what has been found in the same study but with different group [33]. Results from that Norwegian study showed that those who ate less seafood and dairy products had significantly lower urine iodine compared to those who ate more than 60 g of seafood and consumed 1.5 dL dairy products daily [33]. 

The distribution of intake was assessed to determine the proportion of the population below the EAR [10]. If the distribution of habitual iodine intake in a population is known, the percentage of values below EAR of 95 μg/day will give a more reliable estimate of the proportion of the studied population that is truly iodine deficient. Around 30% of males and 41% of females had estimated intakes below the EAR of 95 μg/day. However, our estimated iodine intake was based on one-day assessment of iodine excretion only and information about the day-to-day intra-individual variation in iodine intake is lacking. Studies indicate that when comparing distribution curves of one day and habitual iodine intake, the data are less spread out and the median moves towards higher values for habitual iodine intake [25]. We would therefore expect the real proportion of iodine deficient individuals in our study to be less than 30% of the males and 41% of the women. Still, both the EAR cut-point method and the criteria endorsed by WHO for the assessment of optimal iodine status, indicate that iodine intake in the Somali population is insufficient. 

Despite that the majority of the participants in our study had estimated iodine intake or UIC below the recommended values, all participants had serum free T3 and T4 within the laboratory reference range. Elevated TSH (above 4.0 mU/L) was only found in seven participants and generally there was no association between UIC, UIE and any markers of thyroid function.

## 13. Study Limitations and Strengths

To our knowledge, this is the first population-based study among healthy immigrants in which 24-h urine sampling has been completed. There was also user involvement in the entire study process, which led to high participation and low dropout rate. The other strength of this study was that data collection was conducted during the whole year, and it is known that milk and milk products have a higher iodine concentration during winter months than summer ones due to an increased use of fortified cattle feed during the winter period. In this study, we did not find any seasonal variation in iodine status. 

The study has some limitations. The participants who were eligible for the study but did not participate and those who did not collect 24-h urine might lead to selection bias. In addition, the participants were only selected from one district in Oslo. On the other hand, comparison of the education levels and age distribution of included participants to data from Statistics Norway suggests that the participants included in the present study are representative of adults with Somali background living in Norway.

The lack of associations between factors such as consumption of milk, milk products, fish, egg and supplements and UIC show that, even if we used carefully selected food frequency questions, these questions probably did not provide reliable information on iodine intake in this group. We were not able to calculate the iodine content of these food commodities due to the lack of portion sizes and it is therefore difficult to determine the real sources of iodine for Somali immigrants in Norway. In addition, the intra-individual UIC values vary so much that it made it harder to find relationships.

Although we used methods that are recognised as the gold standards for iodine measurements, the 24-h urine collection was measured only once. Because of day-to-day variation, multiple measurements is optimal [34], however, conducting repeated measurements was not feasible in this study. 

## 14. Conclusions

The findings of this study indicate insufficient iodine status in the Somali population regardless of gender, socio-economic background, age and season. Calculations of iodine intake should be made in different groups of the population and the national authorities should initiate measures that ensure adequate iodine status throughout the population, and especially in vulnerable groups.

## Figures and Tables

**Table 1:** Characteristics of the study population.

	All	Females	Males
Gender (*n*)	169	91	78
Age, years ((mean (SD))	40 (11)	39 (10)	42 (11)
Education, % (*n*)			
Primary or less than primary school	49 (82)	63 (57)	35 (25)
Secondary school	28 (48)	24 (22)	33 (26)
High school or university	23 (39)	13 (12)	32 (27)
Civil status %, (*n*)			
Married	68 (115)	63 (57)	74 (58)
Non-married *	32 (54)	37 (34)	26 (20)
Time lived in Norway, years (mean (SD))	13.6 (7.5)	12.3 (5.8)	13 (6.6)

* Divorced or not married.

**Table 2:** Mean, median and interquartile ranges of 24-h urine volume, 24-h urine iodine concentration (UIC), 24-h iodine excretion (UIE) and thyroid hormones split by sex.

	All			Females			Males		
	Mean (SD)	Median	25th, 75th Percentiles	Mean (SD)	Median	25th, 75th Percentiles	Mean (SD)	Median	25th, 75th Percentiles
24-h urine volume (L)	1.93 (0.7)	1.9	(1.4, 2.5)	1.83 (0.7)	1.75	(1.4, 2.3)	2.06 (0.78)	2	(1.4, 2.6)
24-UIC (µg/L)	73.6 (44.5)	62.5	(38, 100)	71.7 (42.5)	62.5	(50, 90)	75.8	62.5	(38, 100)
24-h UIE (μg/day) *	127.4 (76)	114.4	(80, 155)	119 (70.7)	105	(74, 154)	137.2 (80.8)	122.5	(83, 159)
24-h estimated iodine intake (μg/day)	141 (84)	124	(89, 172)	132 (79)	114	(83, 70)	152 (90)	133 (92, 175)	
**Thyroid hormones ^†^**									
TSH, mU/L (*n* = 150)	2,1 (1.1)	1.9		2.0 (1.0)	1.8		2.1 (1.1)	2	
fT3, pmol/L (*n* = 149)	5.1 (0.6)	5.1		4.9 (0.5)	4.9		5.3 (0.5)	5.3	
fT4, pmol/L (*n* = 147)	15.0 (2.1)	14.9		14.6 (2.3)	14.6		15.5 (1.8)	15.5	

* UIC × 24-h urine volume ^†^ Reference range for Thyroid stimulating hormone (TSH) (0.20–4.0 mU/L), for fT3 (3.5–6.5 pmol/L), for fT4 (11.0–23.0 pmol/L).

**Table 3:** Categorization of iodine deficiency according to WHO criteria.

Median UIC (μg/L) *			
<20 Severe iodine deficiency			
20–49 Moderate iodine deficiency			
50–99 Mild iodine deficiency			
100–199 Adequate iodine nutrition			
200–299 Above requirements			

* WHO criteria for adult populations [8].

**Table 4:** Food intake patterns.

Milk and Milk Products, % (*n*)	All	Males	Females
<1 cup/day	28.3 (47)	18.2 (14)	37.1 (33)
1 cup/day	39.2 (65)	41.5 (32)	36.9 (33)
>1 cup/day	32.5 (54)	40.3 (31)	26.0 (23)
Fish *, % (*n*)			
<1 time/week	24 (41)	26 (20)	23 (21)
1–2 times/week	40 (67)	35 (27)	44 (46)
3–4 times/week	27 (46)	29 (22)	24 (24)
>5 times/week	8 (14)	10 (8)	7 (6)
Eggs, % (*n*)			
Never or <1 time/week	38 (63)	41 (32)	34 (31)
1–3 times/week	53 (89)	48 (37)	57 (52)
4–6 times/week	5 (9)	4 (3)	7 (6)
1 time/day	4 (7)	7 (5)	2 (2)
Salt, % (*n*)			
Never or <1 time/week	85 (143)	86 (66)	85 (94)
1–2 times/week	5 (8)	5 (4)	4 (4)
3–4 times/week	4 (6)	4 (3)	3 (3)
Daily	6 (11)	5 (4)	8 (7)

* Fatty fish.

## Abbreviations

FFQ	Food frequency questionnaire
fT3	Free fraction of triiodothyronine
fT4	Free fraction of thyroxine
IDD	Iodine deficiency disorders
UIC	Urinary iodine concentration
UIE	Urinary iodine excretion
TSH	Thyroid stimulating hormone
